# Salivary miR-34a Exhibits State-Dependent Dysregulation Across Normal Oral Mucosa, Premalignant Lesions and Oral Squamous Cell Carcinoma

**DOI:** 10.3390/genes16121495

**Published:** 2025-12-15

**Authors:** Iphigenia Gintoni, Stavros Vassiliou, Myrto Kardara Bellou, Athanasios Balakas, Nikolaos Lefantzis, Veronica Papakosta, George P. Chrousos, Christos Yapijakis

**Affiliations:** 1 Unit of Orofacial Genetics, 1st Department of Pediatrics, School of Medicine, National Kapodistrian University of Athens, “Aghia Sophia” Children’s Hospital, 115 27 Athens, Greece; 2 University Research Institute of Maternal and Child Health and Precision Medicine, National Kapodistrian University of Athens, Choremion Laboratory, “Aghia Sophia” Children’s Hospital, 115 27 Athens, Greece; chrousge@med.uoa.gr; 3 Department of Oral and Maxillofacial Surgery, School of Medicine, National Kapodistrian University of Athens, Attikon Hospital, 124 62 Athens, Greece; stvasil@med.uoa.gr (S.V.); abalakas63@gmail.com (A.B.); vpapakosta71@gmail.com (V.P.)

**Keywords:** oral cancer, OSCC, precancerous lesions, oral potentially malignant disorders, OPMD, epithelial dysplasia, microRNA, miR-34a-5p, biomarker, liquid biopsy, presymptomatic detection

## Abstract

Background: Oral squamous cell carcinoma (OSCC) is a highly aggressive neoplasm characterized by grim survival outcomes, despite significant therapeutic advances. Mortality rates (up to 70%) have remained unaltered for decades, predominantly due to profound diagnostic delays. These derive from the asymptomatic nature of the early stages of oral carcinogenesis and the emergence of dysplastic areas in previously benign lesions, acting as the bridge to malignant transformation. Hence, the establishment of reliable salivary biomarkers is crucial for non-invasive OSCC detection, even from the premalignant stage of dysplasia. Based on our previous bioinformatic research identifying stage-specific miRNAs throughout OSCC progression, which yielded miR-34a-5p as the most significant, we aimed to experimentally investigate its role in oral oncogenesis and explore its stage-reflecting biomarker potential for liquid biopsy. Methods: The expression of miR-34a was evaluated using quantitative real-time PCR in saliva samples from 9 patients with oral premalignant dysplastic lesions, 10 patients with OSCC and 10 healthy controls. The diagnostic accuracy of miR-34a expression profiles was assessed using ROC-curve analyses. Results: The expression of salivary miR-34a differed significantly across the studied groups, demonstrating a steep decrease in the presence of epithelial premalignant dysplasia, significant upregulation in OSCC and intermediate levels in normal oral mucosa (*p* < 0.001). The ROC results indicate strong diagnostic performance for the detection of oral dysplasia (AUC = 0.93; *p* < 0.001), OSCC (AUC = 0.77; *p* = 0.01) and excellent accuracy for the discrimination between premalignant and OSCC lesions (AUC = 0.98; *p* < 0.001). Conclusions: Our findings reveal a state-dependent dysregulation of miR-34a in oral carcinogenesis, suggesting its complex role as a pathogenetic agent that allows for malignant transformation through its diminished expression, and as a secondary reactive mechanism attempting to suppress tumor development. Salivary miR-34a holds great, stage-specific diagnostic potential, thereby reflecting the health state of oral mucosa in real time.

## 1. Introduction

Oral cancer constitutes the 6th most prevalent type of malignancy worldwide and is histologically represented by oral squamous cell carcinoma (OSCC), which accounts for over 95% of neoplasms of the oral region, with more than 350,000 new cases reported each year [1,2,3]. OSCC arises from malignant cells located in the stratified squamous oral epithelium and typically manifests as cancerous lesions of the lips, tongue, gingiva, buccal mucosa, soft and hard palate, retromolar trigone and the floor of the mouth [4,5,6,7].

OSCC tumors exhibit highly aggressive characteristics and present with strong potential for lymph-node infiltration and metastases that occur in up to 60% of cases, as well as with notably high relapse rates [1,6,8,9]. These traits place OSCC as the fifteenth leading cause of death worldwide, accounting for up to 3–10% of cancer-related mortality [8,10]. OSCC pathogenesis has been attributed the interaction of well-established genetic and environmental factors. The latter include smoking, alcohol consumption, betel quid chewing, poor oral hygiene and infection with oncogenic viral agents, such as types of human papilloma virus (HPV), which are present in up to 34% of premalignant and SCC oral lesions [11,12].

The histopathological progression from normal mucosa to OSCC occurs through the multistep process of oral carcinogenesis, which encompasses the intermediate sequential stages of hyperplasia, dysplasia, in situ carcinoma and the early invasion of OSCC cells, with dysplasia serving as the bridge between the precancerous and cancerous states [13,14,15]. This process may remain completely asymptomatic until apparent OSCC formation or it may present with the occurrence of precursor manifestations, also known as OPMDs (oral potentially malignant disorders) [13,16]. OPMDs comprise lesions such as leukoplakia and erythroplakia, which may exhibit dysplastic areas that highly predispose them to malignant transformation. OSCC development is preceded by premalignant lesions in around 64% of cases, whereas patients with oral epithelial dysplasia face an approximately 45-fold higher risk for OSCC development within two years [5,16,17,18,19].

Despite significant advances in the fields of histopathological diagnosis and treatment, OSCC survival rates have remained alarmingly low for decades, ranging from 30 to 50% in the first three years, whereas 5-year survival rates are estimated at approximately 50–60% [1,9,20,21,22]. The notably unfavorable prognosis of OSCC is primarily attributed to the significant diagnostic delay that is observed in the vast majority of patients, leading to OSCC detection at late stages (III/IV), when lymphatic infiltration and metastases are already present [7,13]. This diagnostic delay stems from the asymptomatic nature of the early stages of oral carcinogenesis, such as hyperplasia and dysplasia, and, even in the case of OPMDs, the presence of dysplastic areas that renders them premalignant is not always evident at the onset [13,15,19]. Hence, the need for presymptomatic non-invasive diagnosis has been highly underscored in the context of OSCC, both for subclinical malignancy and precancerous epithelial dysplasia, as well as for dysplastic OPMDs with increased potential for malignant transformation. To that end, the establishment of reliable biomarkers has been repeatedly proposed, which, in turn, calls for a deeper understanding of the pathogenetic mechanisms of OSCC and the identification of disease-specific molecular signatures [3,6,15,17].

The anatomical positions of OSCC tumors and OPMDs, as well as saliva’s direct contact with the lesions and ease of collection, render it as the most promising body fluid for liquid biopsy [23,24]. Saliva is a rich source of extracellular vesicles (EVs) that are secreted from epithelial cells and contain high amounts of stable and quantifiable molecules, such as microRNAs (miRNAs) [3,25,26,27]. MiRNAs comprise a class of endogenous small, single stranded, non-coding RNAs, which play a crucial role in the post-transcriptional regulation of gene expression through the binding of their seed sequence on complementary sites located on the 3′untraslated regions (3′-UTRs) of their target mRNAs, thereby inhibiting translation or promoting indirect mRNA degradation [1,28,29]. The dysregulation of miRNA expression plays a key pathogenetic role in various diseases and can be highly reflective of pathological conditions. In the context of OSCC alone, over 230 miRNA molecules have been identified as upregulated or downregulated and thus many of them have been proposed as promising biomarkers for liquid biopsy, through blood and saliva evaluation. However, despite the plethora of available expressional data, none have been experimentally validated as disease- and stage-specific, therefore allowing for the reliable detection of OSCC and premalignant dysplasia [6,13].

Recently, our group developed a combinatorial genetic/epigenetic bioinformatic model for the identification of OSCC-specific miRNAs, which simultaneously target the majority of implicated oncogenes and tumor suppressor genes, while exhibiting a reverse dysregulation pattern in OSCC-derived specimens. This approach refined the initial dataset of more than 230 dysregulated molecules down to 5 miRNAs (miR-34a-5p, miR-155-5p, miR-124-3p, miR-1-3p and miR-16-5p), which were yielded as the most representative of the pathology [6]. In 2024, by building upon this methodology, we developed an award-winning respective model with stage-specificity [13], which incorporated human miRNA expressional data from OSCC-derived salivary and tissue specimens across the distinct genetic signature of each stage of oral carcinogenesis, including hyperplasia, dysplasia and the early invasion of OSCC cells, in accordance with our group’s highly distinguished experimental model of sequential oral oncogenesis in Syrian hamsters [14,30]. The disease-specific approach yielded miR-34a-5p (miR-34a) as the most specific miRNA for OSCC, since it simultaneously regulates the expression of all 15 predominantly implicated oncogenes and 5 tumor suppressor genes while exhibiting bidirectional dysregulation in OSCC, thus acting both as a potent oncomiR and a tumor suppressor miRNA [6]. On the other hand, the stage-specific model that incorporated distinct upregulated and downregulated gene-panels for each stage of oral oncogenesis, according to the aforementioned mammalian system, as well as OSCC-derived miRNA expression data and mRNA/miRNA interactions, also yielded miR-34a as the most significant molecule for each sequential stage. In fact, its significant downregulation was predicted in all sequential stages of oral oncogenesis (hyperplasia, dysplasia and the early invasion of OSCC cells). However, the initial malignant stage of early invasion was distinguished as it also encompassed the overexpression of miR-34a, thereby suggesting a more complex and possibly stage-specific role that could not be further elucidated at the time, since the incorporated expressional data were derived from OSCC specimens, which typically encompass areas of all preceding histological stages within the same tumor mass [13].

In the present study, we aimed to experimentally evaluate the bioinformatically predicted stage-specific involvement of miR-34a-5p in oral carcinogenesis through the quantitative assessment of its expression in saliva specimens collected from three groups, corresponding to different histopathological states of the oral epithelium. These included patients with oral dysplastic lesions, OSCC patients, as well as healthy controls of comparable age and gender. The expression profiles of salivary miR-34a were furtherly assessed for their accuracy in detecting each pathological state, as well as in discriminating between OSCC and premalignant dysplastic lesions.

## 2. Materials and Methods

### 2.1. Sample Collection

The present study was approved by the Bioethics Committee of the Department of Oral and Maxillofacial Surgery (1-22-11-2021) and the Scientific Council (A7-08-02-2022) of the “Attikon” University General Hospital. Saliva samples were collected from patients with OSCC, patients with oral premalignant dysplastic lesions and from healthy controls. Eligible patients were individuals with a clinical and histological diagnosis of OSCC or dysplastic premalignant lesions, which were active at the time of sample collection, without any concurrent oral lesions of different etiology or any prior treatment and with no history of recent infections or other systemic inflammatory diseases. The premalignant cohort was exclusively defined by the presence of oral epithelial dysplasia, regardless of additional clinicopathological characteristics, such as lesion subtype or grade. This criterion was applied to ensure experimental comparability with our previously developed model for stage-specific miRNA identification in oral oncogenesis, which incorporated genetic expression data solely according to the histological stage of the oral epithelium, without incorporating additional histological parameterization. The control group comprised healthy individuals of respective age and gender who had no history of OSCC or OPMDs, recent infections or systemic inflammatory diseases. All enrolled participants provided informed written consent for the collection of their demographic information and clinicopathological data, as well as for the collection of salivary samples and the performance of subsequent molecular analyses.

From each participant, 2 mL of whole unstimulated saliva were atraumatically collected using the Saliva RNA collection and preservation devices (Norgen Biotek, Thorold, ON, Canada), including the subsequent addition of the manufacturer’s respective aqueous buffer to the collected samples, allowing for cellular lysis and RNA preservation. All participants had refrained from food or water consumption for at least one hour prior to sample collection and were instructed to rinse their mouths with water 15 min before the procedure. The obtained saliva samples were visually inspected to confirm the absence of major blood contamination and were manually agitated to ensure homogenization. Finally, the specimens were stored at 4 °C until further processing.

### 2.2. Extraction of Salivary Total RNA

Total RNA was extracted from 250 μL of each salivary sample, which was previously mixed with 150 μL of sterile 1xPBS (pH = 7–7.5) (Capricorn Scientific, Ebsdorfergrund, Germany). The extraction of RNA was conducted using the Saliva/Swab RNA Purification Kit (Norgen Biotek, Thorold, ON, Canada), according to the manufacturer’s protocol for preserved saliva. The RNA was eluted in half of the recommended volume to maximize the concentration of obtained miRNAs for subsequent analyses. The RNA samples were evaluated for purity and concentration (ng/μL) using the BioSpec-nano Spectrophotometer for Life-Science (Shimadzu Corporation, Kyoto, Japan). The studied samples demonstrated OD260/280 ratios ranging from 1.94 to 2.06, thus indicating high purity and suitability for reliable downstream analysis. The RNA was reverse-transcribed to complementary DNA (cDNA) directly after extraction and subsequent normalization across sample concentrations, which was performed to ensure equivalency for comparative analysis.

### 2.3. cDNA Synthesis and Quantitative Real-Time PCR for miR-34a Expression Profiling

The synthesis of miRNA-specific cDNA was performed based on polyadenylation, in which a poly(A) tail was added to the 3′end of each mature miRNA, as well as reverse transcription of the RNA, through the use of oligo-dT primers, in a single reaction of 10 μL. The latter contained 2 μL of 5xRT SYBR-Green Reaction Buffer, 2 μL of RNA template, 1 μL of 10xRT Enzyme-Mix and 5 μL of RNase-free water from the miRCURY LNA RT Kit (Qiagen, Hilden, Germany). The reaction was performed on a Gradient Thermal Cycler (Takara Bio, Shiga, Japan), with the thermocycling protocol involving an incubation step of 60 min at 40 °C, followed by inactivation at 95 °C for 5 min and cooling at 4 °C for another 5 min. The obtained cDNA samples were processed the following day after overnight storage at −20 °C.

In order to eliminate possible variability arising from the use of a single reference gene across three distinct histopathological states, the expression of salivary miR-34a (miRBase: MIMAT0000255) was evaluated by absolute quantification. The latter was performed using a standard curve of high linearity (R^2^ = 0.992) and efficiency (96%), produced by serial 2-fold dilutions of reverse-transcribed synthetic oligonucleotides corresponding to mature miR-34a (including matching length, GC% and molecular weight). Reverse transcription of the synthetic molecules was conducted under the same protocol as the studied salivary miRNA (LNA series, Qiagen, Hilden, Germany).

The quantification of miRNA levels (copies/μL) was performed using quantitative Real-Time PCR (RT-qPCR) on a LightCycler 480II platform (Roche, Basel, Switzerland), employing the SYBR-Green fluorescence detection system, in which fluorescence increases proportionally to the accumulation of double-stranded DNA during each amplification cycle, allowing for SYBR-Green binding and signal detection. The term “copies/μL” denotes the concentration per microliter of the qPCR reaction mixture as derived from the standard curve for absolute quantification. Each 10 μL reaction consisted of 5 μL of miRCURY SYBR-Green Master Mix, 4 μL of cDNA template and 1 μL of miRCURY LNA miRNA PCR Assay for the detection of miR-34a (Qiagen, Hilden, Germany). The qPCR thermal protocol incorporated a heat activation step for 10 min at 95 °C, followed by 45 cycles of 95 °C for 15 s and 60 °C for 1 min, as well as a melting-curve analysis final cycle ranging from 60 °C to 95 °C (0.03 °C/s rate) with continuous fluorescence acquisition.

Threshold cycle (Ct) values were obtained using the Absolute Quantification/2nd Derivative Max analysis in the LightCycler 480II software (Roche, Basel, Switzerland). The experimental protocol incorporated 45 amplification cycles to technically ensure the reliable detection of possibly low-abundance targets. However, a cutoff threshold of 40 cycles was applied to ensure reliability by avoiding non-specific signals. No amplification signals approached the cutoff threshold, thereby confirming the robustness and sensitivity of the assay.

### 2.4. Statistical Analysis

Statistical analyses were performed using version 29.0.2.0 of IBM SPSS Statistics (IBM Corp., Armonk, NY, USA). Normality was assessed using the Shapiro–Wilk test for continuous values, in accordance with the sample size (<50). Comparisons between independent groups were conducted using the Mann–Whitney U non-parametric test, with respect to the distribution of data. Comparisons between more than two groups were conducted using the Kruskal–Wallis test, whereas the Jonckheere–Terpstra test was also applied for comparisons of ordered groups and subsequent pairwise analyses. All presented, comparative figures include error bars for each group, illustrating the variability of observed values around the means. An a priori power analysis (one-way ANOVA, α = 0.05; *n* = 29) was performed and indicated that, with the enrolled groups (10 OSCC, 9 patients with premalignant lesions and 10 controls), the present study demonstrated 99.7% power to detect large effects (Cohen’s f = 1.0).

Individual Receiver Operating Characteristic (ROC) analyses were performed for each pair of study groups in order to evaluate the diagnostic accuracy of salivary miR-34a-5p in detecting each pathological state and distinguishing it from normal oral epithelium, as well as in discriminating between OSCC and premalignant dysplastic lesions. For each analysis, the corresponding ROC curve was generated, followed by the calculation of the Area under the Curve (AUC) and the respective *p* value, indicating the diagnostic performance of the potential biomarker. The level of statistical significance was set at *p* < 0.05 (two-tailed).

Multivariable Statistical analyses were not performed in the present proof-of-concept study, despite the availability of clinicopathological data, as its design focused exclusively on oral histopathological status (normal epithelium, presence of dysplastic regions and OSCC) without any additional covariates. Additionally, due to the limited size of each group, such analyses were not pursued so as not to compromise statistical power and to equally explore the stage-specific expression patterns predicted by the aforementioned bioinformatic model, which was based solely on histological stage transitions of oral carcinogenesis, rather than on additional clinicopathologic parametrization.

## 3. Results

### 3.1. Clinicopathological and Demographic Characteristics of the Studied Groups

A total of 29 participants were enrolled in the present study, including 10 patients with OSCC, 9 patients with premalignant oral lesions and 10 healthy controls. Table 1 provides an overview of the demographic characteristics (age and gender) across the three studied groups and the relevant clinicopathological data of the enrolled patients, including histological diagnosis, the anatomical site of the oral lesions, as well as tumor grade and invasiveness status (applicable to OSCC patients).

A comparison of age among the three groups was performed using the Kruskal–Wallis test, revealing no statistically significant differences (*p* = 0.455). Gender distribution was also comparable (Chi-Square test, *p* = 0.996). The lesion sites included the retromolar trigone, floor of mouth, buccal mucosa, gingiva, alveolar ridge, lower lip and lateral boarder and ventral surface of the tongue. In terms of tumor grading, the OSCC group encompassed well-differentiated, moderately differentiated and poorly differentiated cases, thereby capturing the full histopathological spectrum, whereas the vast majority of OSCC patients (90%) presented with highly invasive tumors, which infiltrated adjacent soft tissues, perineural spaces or bone structures (Table 1). The included premalignant lesions, mainly located on buccal mucosa and the lower lip, demonstrated dysplastic areas, accompanied by hyperplastic components and inflammatory infiltrates.

### 3.2. Expression of Salivary miR-34a in OSCC, Premalignant Lesions and Healthy Controls

**Figure 1:**
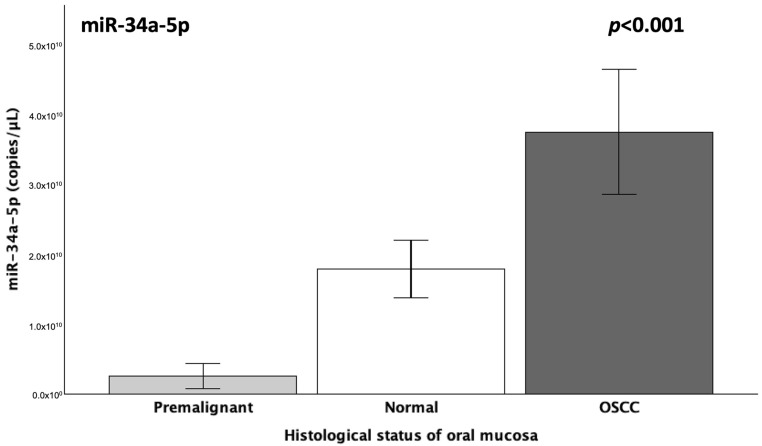
Graphs depicting the mean expression (copies/μL) of salivary miR-34a-5p across the three studied groups, which correspond to distinct histopathological states of oral epithelium: premalignant dysplastic lesions, normal oral mucosa and differentiated OSCC (*p* < 0.001).

The quantification results of salivary miR-34a-5p expression (copies/μL) differed significantly among the studied groups, according to the Kruskal–Wallis test (*p* < 0.001). This was furtherly supported by the Jonckheere–Terpstra test, which detected a highly significant monotonic increase in miR-34a expression across the groups, following the order premalignant lesions < normal mucosa < OSCC (*p* < 0.001), as illustrated in Figure 1.

**Figure 2:**
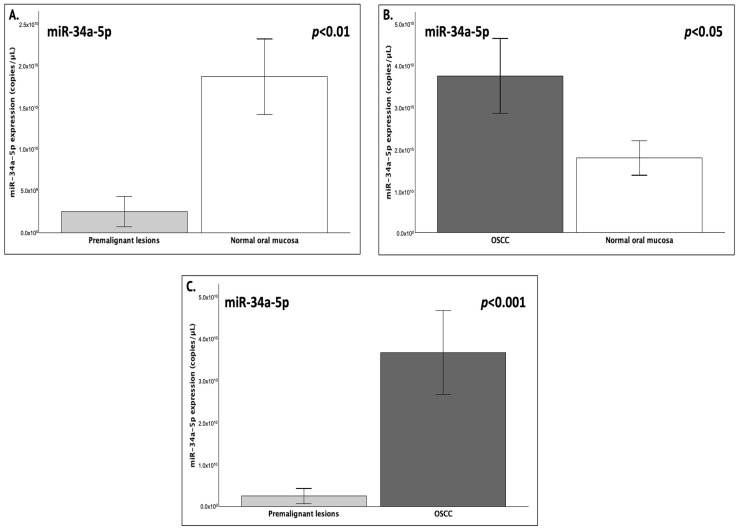
The graphs depict the mean expression (copies/μL) of salivary miR-34a-5p between (**A**) patients with premalignant dysplastic lesions and healthy controls (*p* = 0.001), (**B**) patients with OSCC and healthy controls (*p* = 0.041) and (**C**) patients with premalignant dysplastic lesions and patients with OSCC (*p* < 0.001). Statistical significance was set at *p* < 0.05.

Pairwise comparisons, which were additionally confirmed with Mann–Whitney U tests for all groups, indicated that all pairs differed significantly in terms of miR-34a expression (*p* < 0.05) (Figure 2). In greater detail, salivary miR-34a expression exhibited an 86% decrease in the presence of premalignant oral lesions compared to normal mucosa (FC = 0.14; *p* = 0.001) (Figure 2). Conversely, the salivary samples of OSCC patients showed a significant 110% increase in miR-34a levels compared with healthy controls (FC = 2.10; *p* = 0.041). Finally, when comparing the two patient groups, a 14.7-fold increase was observed in the miR-34a levels was observed in OSCC saliva samples compared to those from precancerous dysplastic lesions (FC = 14.7; *p* < 0.001).

### 3.3. Diagnostic Performance of Salivary miR-34a (ROC Analysis)

**Figure 3:**
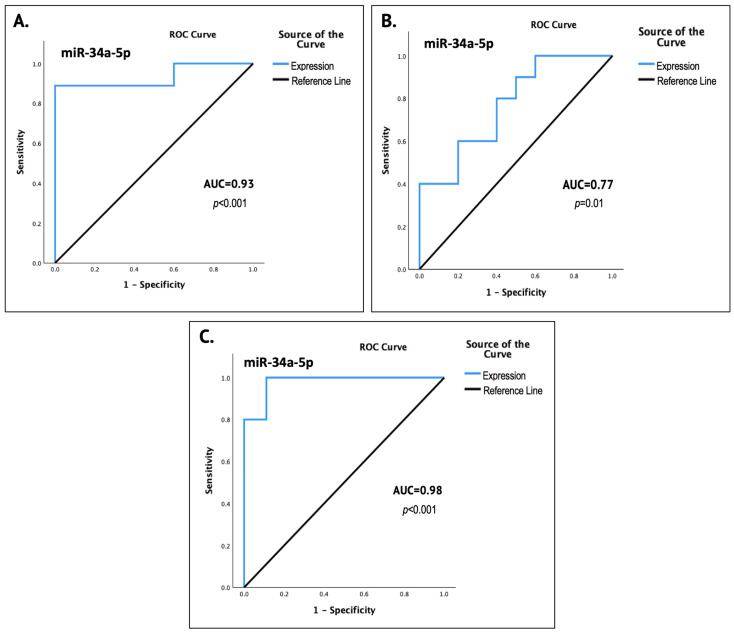
The graphs depict the results for each individual ROC analysis that was performed to evaluate the diagnostic accuracy of salivary miR-34a-5p for the distinguishing of (**A**) oral epithelial dysplasia from normal mucosa, (**B**) OSCC from normal oral epithelium and (**C**) the discrimination between OSCC and premalignant dysplastic lesions. Each graph incorporates the corresponding ROC curve, AUC value and the respective *p* value, indicating the diagnostic performance of miR-34a expression in salivary specimens. The level of statistical significance was set at *p* < 0.05.

The expression levels of salivary miR-34a demonstrated significant diagnostic performance for detecting each pathological state, as well as discriminating between premalignant dysplastic lesions and OSCC, with AUC values ranging from 0.77 to 0.98 (*p* < 0.05), as determined by the respective ROC curve analyses presented in Figure 3.

Specifically, for the detection of premalignant lesions and their discrimination from normal oral mucosa, the ROC curve analysis yielded an AUC of 0.93 (95% CI: 0.80–1.00, *p* < 0.001), indicating a significantly high diagnostic accuracy of salivary miR-34a expression. The respective analysis for distinguishing OSCC lesions from normal epithelium demonstrated adequate diagnostic capacity, with an AUC of 0.77 (95% CI: 0.56–0.98, *p* = 0.01). Finally, the ROC curve analysis between the groups of OSCC and premalignant lesions revealed excellent diagnostic performance, with an AUC of 0.98 (95% CI: 0.92–1.00, *p* < 0.001), reflecting an approximately 98% probability of valid discrimination between precancerous dysplastic lesions and OSCC, solely based on miR-34a quantification in saliva.

## 4. Discussion

OSCC, which represents over 95% of oral cancer cases, is a highly aggressive neoplasm with strong tendencies for lymph node infiltration, as well as distant metastases, thus ranking as the fifth leading cause of death worldwide [1,6,8,9]. Despite extensive research and significant therapeutic advances, OSCC continues to be associated with grim survival outcomes, as the accompanying mortality rates reach up to 70% and remain unaltered for decades [1,9]. The extremely high OSCC-related mortality is predominantly attributed to delayed diagnosis in most patients. The latter derives from the asymptomatic nature of the early sequential stages of oral carcinogenesis (hyperplasia, dysplasia and early invasion), subclinical malignancy and the emergence of precancerous dysplasia in previously benign oral lesions, which acts as the “histological bridge” to malignant transformation [5,13,14,15]. Hence, the establishment of reliable biomarkers is of paramount importance for the non-invasive, timely diagnosis of OSCC or even for presymptomatic detection from the earliest stage of oral dysplasia. Saliva, the primary source of tumor-derived EVs, represents a promising candidate for liquid biopsy through the evaluation of distinct patterns of miRNA dysregulation that reflect the pathohistological stage of the oral epithelium [13,24,25,27].

Recently, we developed a bioinformatic model for the identification of stage-specific miRNAs for oral carcinogenesis, among the vast pool of >230 molecules that have been experimentally reported as dysregulated in OSCC-derived tissues and saliva specimens [13]. This was performed by incorporating significant OSCC-derived miRNA expression patterns, as well as gene expression data from our group’s mammalian model of sequential oral oncogenesis in the form of customized panels of characteristically upregulated or downregulated genes for each stage. This allowed for the evaluation of miRNA OSCC-derived expression patterns across each stage’s signature dysregulation of key OSCC-related genes, while considering that the final tumor encompasses all preceding sequential stages, along with malignant regions [13,14,30]. Our analyses yielded miR-34a-5p as the most specific molecule for each stage of oral oncogenesis (precancerous hyperplasia, dysplasia and malignant early invasion), since it has been shown to epigenetically regulate the expression of every implicated gene, while it has also demonstrated an inverse expression profile in OSCC compared to its target-genes’ stage-specific dysregulation. MiR-34a was predicted to significantly decrease in the precancerous stages of hyperplasia and dysplasia, whereas a dual pattern was observed for the initial malignant stage of early invasion, consistent with both the overexpression and downregulation of miR-34a. Therefore, a more complex, possibly state-dependent role was proposed; however, the available experimental data in the OSCC-related literature were not sufficient for its elucidation [13].

In the present study, building upon our previous bioinformatic research, we aimed to experimentally investigate the putative stage-specific role of miR-34a-5p in oral oncogenesis through the quantification of its expression in the salivary samples of three groups, corresponding to key histopathological stages of malignant progression. This included patients with premalignant dysplastic oral lesions, patients with OSCC and gender- and age-matched controls with clinically normal oral mucosa. The definition of the premalignant group was based exclusively on the presence of dysplastic regions was intentional, as dysplasia represents the bridge stage towards malignant transformation. Additionally, the fact that dysplastic and inflammatory areas are known to coexist in both dysplastic and OSCC lesions ensured that any observed differential expression of miR-34a between the premalignant and OSCC groups would indeed reflect state-dependent effects, rather than incidental inter-lesional variability.

Our guiding hypothesis of state-dependent dysregulation was indeed experimentally confirmed in the current exploratory setting, as salivary miR-34a expression differed significantly across the studied groups, demonstrating a nadir in the presence of epithelial premalignant dysplasia, a significant increase in light of OSCC lesions and intermediate levels in normal oral mucosa (*p* < 0.001) (Figure 1). The results of the ROC curve analyses significantly underlined the potential of miR-34a as a state-reflecting salivary biomarker, as they indicated strong diagnostic performance, with an AUC of 0.93 (93%) for the non-invasive detection of epithelial dysplasia (*p* < 0.001) and 0.77 (77%) for OSCC (*p* = 0.01), as well as possibly excellent accuracy in discriminating between premalignant and OSCC lesions, with an AUC of 0.98 (98%) (*p* < 0.001).

**Figure 4:**
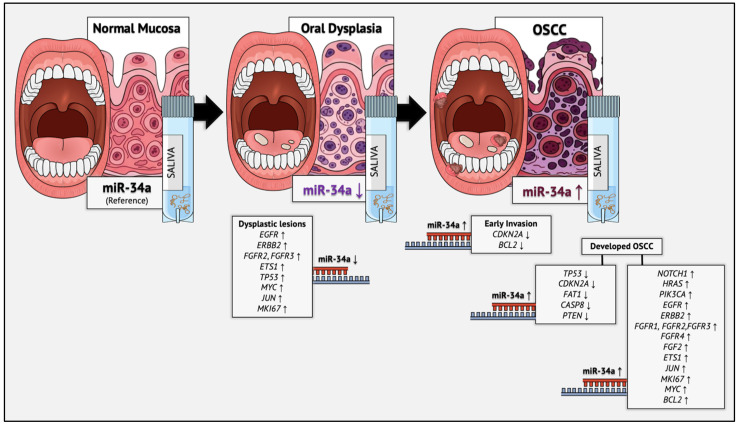
Graphical summary of the dysregulation patterns of miR-34a, which were observed in the present study. According to our findings, salivary miR-34a differed significantly across the studied groups, demonstrating a steep decrease in the presence of epithelial premalignant dysplasia, a significant upregulation in OSCC and intermediate levels for normal oral mucosa (*p* < 0.001). The evaluation of miR-34a levels in saliva exhibited strong diagnostic performance as a potential non-invasive biomarker for the detection of oral dysplasia and OSCC lesions, but also for highly accurate discrimination between premalignant and OSCC lesions. Therefore, our findings revealed state-dependent dysregulation of miR-34a in saliva, thereby suggesting its complex involvement in OSCC development through the establishment of the precursor dysplastic phenotype that is facilitated by its expressional loss, but also its role as a secondary compensating mechanism, attempting to counterbalance OSCC progression and oncogenic signaling. The black horizontal arrows used in the figure represent the sequential transition from each histological stage to its successor in oral carcinogenesis, while the upward and downward arrows indicate the expressional pattern of miR-34a (significant upregulation or downregulation) and also the respective characteristic dysregulation of key genes, which defines the genetic signature of each histological stage, according to the experimental model of sequential oral oncogenesis [13,14,30].

According to our previous bioinformatic research, miR-34a has been found to simultaneously target and negatively regulate the expression of *EGFR*, *ERBB2*, *FGFR2*, *FGFR3*, *ETS1*, *TP53*, *MYC*, *JUN* and *MKI67* genes that are characteristically upregulated in premalignant oral dysplasia (Figure 4), which results in OSCC formation, as yielded from the experimental model of sequential oral oncogenesis [13,30]. In the present molecular study, the 86% reduction in miR-34a expression observed in the group of premalignant dysplastic lesions may reflect impaired regulatory efficiency over the aforementioned target-gene panel, thereby facilitating the establishment of the dysplastic phenotype in the oral epithelium and serving as an indicator of its transition towards a premalignant state. This conclusion is reinforced by its previously reported downregulation in lesions of oral leukoplakia, which often contain dysplastic histological areas [15,25].

Regarding the malignant state of OSCC, our previous analyses yielded highly consistent results that corroborate the present experimental findings. Specifically, miR-34a, which emerged as both the most disease- and stage-specific molecule in oral oncogenesis, has been shown to regulate the expression of all five tumor suppressor genes that are pathologically downregulated in OSCC pathogenesis (*TP53*, *CDKN2A*, *FAT1*, *CASP8* and *PTEN*) [6], as well as the two characteristically downregulated genes that govern the initial malignant stage of early invasion (*CDKN2A* and *BCL2*). On the other hand, it has been shown to normally regulate all fifteen major oncogenes that have been widely recognized to contribute to OSCC progression through their overexpression (*NOTCH1*, *HRAS*, *PIK3CA*, *EGFR*, *ERBB2*, *FGFR1*, *FGFR2*, *FGFR3*, *FGFR4*, *FGF2*, *ETS1*, *JUN*, *MKI67*, *MYC* and *BCL2*) [13,30] (Figure 4).

Hence, the observed 110% increase in salivary miR-34a in the OSCC patient group, compared to healthy individuals, possibly suggests an adaptive compensating response to malignancy. This speculative interpretation is furtherly supported by a plethora of functional studies that underline the inhibitory properties of miR-34a overexpression on OSCC proliferation, oncogenic signaling and metastatic rates [26,31,32,33]. The observed increase in miR-34a in the context of a pronounced inflammatory state, such as malignancy, also corroborates its previously highlighted function as a compensatory epigenetic inflammatory inhibitor [34]. In fact, higher miR-34a levels have been reported as a response to alcohol consumption in OSCC patients, which is known to induce cancer-associated inflammation [35,36]. Finally, the observed miR-34a overexpression in salivary samples from the OSCC group aligns with findings from a study of circulating miRNA profiles in cervical SCC, which places miR-34a among the characteristically upregulated molecules in patients’ serum (*p* < 0.001), thus underlining its possibly reactive role and its potential as a non-invasive diagnostic marker [37].

The majority of published relevant studies have documented the downregulation of miR-34a in OSCC tissue specimens, although upregulation has also been reported, as well as its downregulation in potentially premalignant disorders such as oral leukoplakia [6,13,25,32,38,39,40]. These results are in accordance with the dual role elucidated in the present research. Nevertheless, the significant upregulation of salivary miR-34a that was observed here, in the OSCC group, differs from its usually reported pattern in tissue-based studies. This disparity might be attributable to the profound decrease in miR-34a levels observed in the group of dysplastic lesions, which was 93% lower than in the corresponding OSCC lesions (FC = 0.068; *p* < 0.001) and 86% lower than in healthy controls (FC = 0.14; *p* < 0.001). Specifically, the tumor tissues used as a source of RNA for miRNA studies, inevitably include histological areas that correspond to the preceding stages that led to the pathogenesis of the studied malignancy. Hence, it is possible that in most tissue-based studies, the increase in miR-34a corresponding to the malignant areas might not have been sufficient to counterbalance the profound reduction induced by multiple coexisting dysplastic regions within the tumor, which likely dominate its overall expression profile by imposing downregulation.

Hence, our findings might provide a significant addition to the pile of evidence rendering saliva as the most diagnostically valuable specimen in the context of oral pathologies. This is attributable not only to its non-invasive collection but also to its reduced susceptibility to histological overlap, which is usually inevitable in bulk-tissue samples. The better representation of saliva-based profiling is furtherly supported, as cancer cells are known to produce significantly higher amounts of EVs carrying their miRNA cargo compared to surrounding cells [41]. Therefore, in light of malignancy, salivary miRNAs mostly originate from the cancerous subpopulations of the lesion, providing a clearer “snapshot” of their molecular status [13,27].

This exploratory research represents, to our knowledge, the first experimental elucidation of the stage-specific role of miR-34a across the spectrum of oral carcinogenesis. The findings indicate the potential implication of miR-34a in sequential OSCC pathogenetic mechanisms. Its state-dependent dysregulation possibly renders miR-34a as both an oncogenic and a tumor suppressive molecule through the trajectory of oral carcinogenesis. In fact, miR-34a does not act as a traditional oncomiR but functions similarly to a tumor suppressor gene, as its expressional loss possibly facilitates carcinogenesis through inadequate regulatory control over overexpressed oncogenic targets. The present experimental findings, which are in agreement with our previous bioinformatic predictions, support the proposed role of miR-34a as a tumor suppressor miRNA that has been demonstrated in many functional studies exploring the anticarcinogenic effects of its induced overexpression in OSCC.

The yielded findings also provide a highly promising, state-dependent salivary biomarker that might enable the early detection of OSCC development from the initial stage of oral dysplasia, as well as for the non-invasive, rapid classification of a newly presented lesion as premalignant or cancerous. This could be feasibly incorporated into future clinical dental/medical practice, after the establishment of reliable diagnostic thresholds, through the collection of 0.5 mL of whole saliva, by any clinician, allowing for duplicate analysis (initial assessment and validation). We anticipate that the present research, spanning from multilayered bioinformatic investigation to experimental validation, will pave the way for a more comprehensive exploration of salivary miRNA biomarkers in the context of OSCC, with the ultimate goal of ensuring specificity and contributing to the critical domain of early diagnosis.

### Study Limitations

Despite the adequate statistical power of the present study to detect large effects, as well as the profound statistical significance that characterized the majority of findings, studies including larger cohorts are required for enhanced generalizability and the establishment of miR-34a as a salivary diagnostic biomarker. Such validation is crucial for the reliable definition of expressional thresholds with diagnostic accuracy, which cannot be confidently derived from an exploratory study of limited size. Another limitation concerns the gender ratio of the studied groups. Although OSCC predominantly concerns males, female representation (10–11%) was lower than is typically reported in OSCC cohorts (around 20%) [42,43]. This imbalance in sex distribution is primarily due to the limited size of the premalignant cohort, which was the most restricted and dominated the distribution of the other two groups (OSCC and healthy controls), in order to achieve maximum comparability. Nevertheless, this gender imbalance might be limiting the generalizability of findings in regards to female populations.

## 5. Conclusions

The present findings, in combination with those from our previous bioinformatic studies, provide further insight into the complex role of miR-34a-5p in the context of oral carcinogenesis. Its state-dependent expression in saliva shows a steep decrease in the presence of premalignant dysplastic lesions, significant overexpression in light of OSCC and intermediate levels in normal mucosa. This pattern suggests that miR-34a might act as a key pathogenetic player in OSCC, paving the way for malignant transformation through its expressional loss in oral epithelium. The latter possibly allows for the establishment of a dysplastic phenotype, the bridge to malignancy, through the inadequate regulation of key stage-specific, pathologically overexpressed genes.

Once OSCC has fully occurred, inflammation-driven miR-34a activation might represent a reactive compensatory mechanism to counterbalance tumor progression, perfectly aligning with its functionally established tumor-suppressive properties and its role as an inhibitor of stress-induced inflammation. Hence, the loss of miR-34a might allow for carcinogenesis itself to take place, whereas its secondary upregulation is indicative of a feedback mechanism possibly aiming to restore oral homeostasis. The quantification of miR-34a in salivary samples exhibits immense diagnostic potential in OSCC, with distinct patterns at each stage of oncogenesis, thereby reflecting the health status of oral mucosa in real time, with a minimal amount of specimen required for analysis and validation.

## Figures and Tables

**Table 1:** Summary of the demographic characteristics (age/gender) of the study cohorts and relevant clinicopathological data of the studied patients with OSCC and premalignant lesions. Age and gender parameters were statistically compared among the three groups using appropriate statistical tests. Statistical significance was set at *p* < 0.05. ^1^ Mean (Range); ^a^ Kruskal–Wallis, ^b^ Pearson Chi-Square.

**Variable**	**OSCC Patients** (*n* = 10)				**Patients with Premalignant Lesions** (*n* = 9)			**Controls** (*n* = 10)			***p*-Value**
**Age** (years) ^1^	62.9 (51–80)				64.7 (28–81)			62.7 (53–72)			0.455 ^a^
**Gender**	**Male**		**Female**		**Male**		**Female**	**Male**		**Female**	0.996 ^b^
*n* (%)	9 (90%)		1 (10%)		8 (88.9%)		1 (11.1%)	9 (90%)		1 (10%)	
**OSCC patients**											
**Anatomical site of OSCC lesions** (*n*, %)											
**Tongue (lateral/ventral)**		**Retromolar trigone**			**Floor of mouth**			**Gingiva/Alveolar ridge**			**Lower lip**
4 (40%)		3 (30%)			1 (10%)			1 (10%)			1 (10%)
**OSCC histological grade** (*n*, %)											
**Well-differentiated**				**Moderately differentiated**					**Poorly differentiated**		
1 (10%)				7 (70%)					2 (20%)		
**OSCC invasiveness status** (*n*, %)											
**Invasive**						**Non-invasive**					
9 (90%)						1 (10%)					
**Anatomical site of premalignant lesions** (*n*, %)											
**Lower lip**				**Buccal mucosa**					**Gingiva/Alveolar ridge**		
5 (55.6%)				3 (33.3%)					1 (11.1%)		

## Data Availability

The original data presented in this research are available upon request from the corresponding authors. Certain data may be subject to limited availability, in accordance with confidentiality and privacy protecting protocols or ongoing study conduction.
